# Notes and News

**Published:** 1895-08-24

**Authors:** 


### Notes and News.

(Collected and Communicated.)

The German Government is about to establish a classification distinguishing intelligent children from those of weak intellect attending the schools. Education suitable to the development of the latter will then be secured.

The annual report of the Ingham Infirmary, South Shields, shows a surplus of £111 for the financial year, after providing for all liabilities. There has also been an increase in the capital account, owing to the receipt of several legacies. It is interesting to note that while the annual subscriptions came to £317, the workmen's contributions amounted to £690. This seems to show that the institution is very popular with the class for whose benefit it was founded. We would draw attention, however, to the somewhat careless manner in which the medical report is drawn up. It may be doubted whether there is any advantage in publishing, in a general report, such an analysis of cases as we here find; but if it is published at all it should be drawn up much more carefully than is here the case.

The teaching of hygiene to women has, it is to be feared, often partaken of a somewhat dilletante character. The growing importance of the subject, and the fact that many appointments now open to women require a good knowledge of it, has led the Council of the Bedford College, London (for women), to establish a separate and scientific course of instruction in hygiene. Students will be required to devote themselves for a session or more solely to this and allied branches of science, viz., physiology, bacteriology, chemistry, and physics, which will be taught practically as well as theoretically, and thus they will have the opportunity of obtaining some real knowledge of the subject in its various bearings. As sanitary inspectors and teachers of hygiene under county councils and in schools, many appointments are likely in the future to be open to women who have aptitude for scientific work and are willing to devote themselves to it.

Hospital Saturday has been celebrated in Dover with considerable energy. In addition to the usual collections, a monstre procession of friendly societies and trades, in carnival costume, took place. The money collected amounted to £113.

A large meeting was held on Hampstead Heath on August 18th to protest against the action of the authorities of Hampstead Parish Church in refusing to allow the usual special service to be held therein in connection with the annual demonstration of friendly societies in favour of the North London Hospital for Consumption.

The gradual extension of small-pox in the Metropolis is a matter of considerable concern to other districts maybe far away. London is a centre which is in touch with all parts of the country, and it would be idle to pretend that there is any likelihood of the outbreak, if it should take an epidemic form, being confined within the metropolitan area. Sanitary authorities should then be on their guard, and be prepared with means for immediately isolating any case which may occur in their midst. It is in dealing with the early cases that isolation shows to the greatest advantage, for by immediate isolation an epidemic may often be entirely averted.

An alarming gas explosion took place on August 17th at the Mile End Infirmary. A considerable amount of damage was done. It appears to have been caused by the fracture of a large gas main immediately before its passage, below ground, through the wall of the building. The escaping gas had found its way into the covered trenches along which the water pipes are distributed, and on reaching a light exploded with great violence. This matter is worth noting by hospital authorities, as the placing of water pipes in covered trenches is a rather favourite plan with architects. It is clear that where gas pipes are in the neighbourhood, or, as in some cases, even within the same trench, the danger of leakage and consequent explosion must be carefully reckoned with.

A significant sign of the times in regard to street collections for charitable purposes is to be seen in the result of the twenty-second annual street collection in connection with the Hospital Saturday Fund, which took place on July 13th. This is now found to have realised a sum of £2,965, being a decrease of £1,842 upon that of 1894. The workshop collection on Saturday, the 10th inst., amounted to £6,043. The falling off in the street collections is attributed to the great extension which has recently taken place in this mode of raising money. Charities and institutions of all sorts flood the streets with emissaries, not always the most suitable, who assail the by-passer with such persistency that he hardens his heart and buttons up his pockets. So long as street collections were the outward sign of a great public movement, and were limited to an annual demonstration under the direction of the responsible managers of the Hospital Saturday Fund, all was well; but the whole thing has become commonised. Every week there are collections for some "hospital" or other, or some "fund" which no one ever heard of, and the charitable public, who would give willingly in answer to an occasional well-authorised appeal, are being rapidly educated in the art of saying "No." All this is bad, and, unfortunately, the Hospital Saturday Fund has to pay for excesses of its imitators.

